# Heterozygous disruption of Flk-1 receptor leads to myocardial ischaemia reperfusion injury in mice: application of affymetrix gene chip analysis

**DOI:** 10.1111/j.1582-4934.2008.00269.x

**Published:** 2008-02-08

**Authors:** M Thirunavukkarasu, S Addya, B Juhasz, R Pant, L Zhan, S Surrey, G Maulik, V P Menon, N Maulik

**Affiliations:** a Department of Surgery, Molecular Cardiology and Angiogenesis Laboratory, University of Connecticut Health CenterFarmington, CT, USA; b Cancer Genomics Facility, Kimmel Cancer Center, Thomas Jefferson UniversityPhiladelphia, PA, USA; c Department of Pharmacology, University of DebrecenHungary; d Cardeza Foundation for Hematologic Research, Department of Medicine, Thomas Jefferson UniversityPhiladelphia, PA, USA; e Department of Biochemistry and Biotechnology, Annamalai UniversityTN, India; f Department of Thoracic Surgery, Harvard Medical SchoolBoston, MA, USA

**Keywords:** ischaemia, reperfusion, Flk-1, myocardium, affymetrix gene chip, gene expression

## Abstract

This study addresses an important clinical issue by identifying potential candidates of vascular endothelial growth factor (VEGF) signalling through the Flk-1 receptor that trigger cardioprotective signals under ischaemic stress. Isolated working mouse hearts of both wild-type (WT) and Flk-1^+/−^ were subjected to global ischaemia (I) for 30 min. followed by 2 hrs of reperfusion (R). Flk-1^+/−^ myocardium displayed almost 50% reduction in Flk-1 mRNA as examined by quantitative real-time RT-PCR at the baseline level. Flk-1^+/−^ mouse hearts displayed reduction in left ventricular functional recovery throughout reperfusion (dp/dt 605 *versus* 884), after 2 hrs (*P* < 0.05). Coronary (1.9 *versus* 2.4 ml) and aortic flow (AF) (0.16 *versus* 1.2 ml) were reduced in Flk-1^+/−^ after 2 hrs of reperfusion. In addition, increased infarct size (38.4%*versus* 28.41%, *P* < 0.05) and apoptotic cardiomyocytes (495 *versus* 213) were observed in Flk-1^+/−^ knockout (KO) mice. We also examined whether ischaemic preconditioning (PC), a novel method to induce cardioprotection against ischaemia reperfusion injury, through stimulating the VEGF signalling pathway might function in Flk-1^+/−^ mice. We found that knocking down Flk-1 resulted in significant reduction in the cardioprotective effect by PC compared to WT. Affymetrix gene chip analysis demonstrated down-regulation of important genes after IR and preconditioning followed by ischaemia reperfusion in Flk-1^+/−^ mice compared to WT. To get insight into the underlying molecular pathways involved in ischaemic PC, we determined the distinct and overlapping biological processes using Ingenuity pathway analysis tool. Independent evidence at the mRNA level supporting the Affymetrix results were validated using real-time RT-PCR for selected down-regulated genes, which are thought to play important roles in cardioprotection after ischaemic insult. In summary, our data indicated for the first time that ischaemic PC modifies genomic responses in heterozygous VEGFR-2/Flk-1 KO mice and abolishes its cardioprotective effect on ischaemic myocardium.

## Introduction

Angiogenesis offers enormous potential for therapeutic intervention of many human disorders. Many angiogenesis-related factors are involved in the development of vessels during vascu-logenesis, as well as in the induction of new vessels in response to physiological or pathological stimuli [1]. Angiogenesis and vasculogenesis are regulated predominantly by several different growth factors and their associated receptor tyrosine kinases (RTKs) [2]. Foremost among these are the vascular endothelial growth factor (VEGF) family and its receptors which are essential regulators of angiogenesis and vascular permeability [3]. The central role of VEGF in angiogenesis in health and disease makes it attractive both as a therapeutic target for anti-angiogenic drugs in pathological conditions, such as cancer and as a pro-angiogenic cytokine for the treatment of ischaemic heart disease. VEGF binds to two receptor protein tyrosine kinases, VEGFR1 (Flt-1) and VEGFR2 (Flk-1/KDR), but most of the biological functions of VEGF are mediated via Flk-1 [4]. We demonstrated previously that ischaemic preconditioning (PC) induced angiogenesis in the infarcted myocardium and resulted in up-regulation of several transcription factors (STAT3, Pax-5, nfκb, TFIID, SP1 etc). In addition, PC reduced VEGF-mediated vascular permeability by inhibition of c-Src in the ischaemic preconditioned group, thereby reducing ischaemic injury in a rat myocardial infarction model [5]. However, the mechanism by which activation of VEGFRs elicit these cellular events is not fully understood. Recently, attention has been directed toward studies of VEGF expression and its function in myocardial ischaemia/hypoxia [6–8] and relatively little is known regarding the mechanism of its receptors, Flk-1 and Flt-1. VEGF is the only known ligand for Flk-1, whereas Flt-1 is able to bind placental growth factor in addition to VEGF.

Few embryological studies have demonstrated abundance of Flk-1 in human lung tissues, whereas Flt-1 was abundant in heart, lung and kidneys [9]. Several investigations demonstrated functional difference between Flk-1 and Flt-1 in endothelial cells. In the developing human heart, both receptors were expressed in the myocardial capillaries, and were known to stimulate intracellular calcium flux and VEGF stimulation. Genetically manipulated Flk-1 knockout (KO) (homozygous) studies demonstrated early embryonic death due to inhibition of vasculogenesis, whereas in another study homozygous Flt-1 disruption caused failure to assemble normal vascular channels [10]. Another important observation showed that Flt-1 was expressed in the endothelium of both large and small vessels, whereas Flk-1 expression was restricted only to small vessels [11]. Recent studies demonstrated the myocardial distribution pattern of Flk-1 and Flt-1 after rats were exposed to whole body hypoxia followed by 24 hrs of re-oxygenation. Intense staining was observed along the capillaries in addition to the coronary arteries [7]. We also documented that intensity of staining for both receptors increased significantly in the hypoxia/re-oxygenation group compared to normoxic control. We also documented significant improvement in myocardial function with increased capillary and arteriolar density after induction of survival factors VEGF, Bcl-2 and survivin in the chronic rat myocardial infarction model subjected to ischaemic PC [5].

One of our recent studies indicated reduced beneficial effects of PC in Flt-1 heterozygous KO mice compared to wild-type. This observation may be due to down-regulation of several important genes (obtained by DNA microarray analysis) such as oncogene 1 (Gro1), heat shock proteins, I Kappa B Kinase β (IKKβ), colony stimulating factor (CSF-1) and annexin 7, suggesting the importance of VEGF /Flt-1 receptor signalling during ischaemic PC [12]. To gain a better understanding of the VEGF signalling through its other homologous membrane-spanning high-affinity tyrosine kinase receptor, we performed microarray analysis (Affymetrix Gene Chip Analysis) on Flk-1^+/−^ KO mice subjected to ischaemia reperfusion (IR) and preconditioning followed by ischaemia reperfusion (PCIR) protocols.

The results obtained from this investigation not only defined a high number of up- and down- regulated known and unknown genes in IR and PCIR when comparing wild-type (WT) and KO, but also provided functional network information. We observed many differentially expressed genes after IR (115) and PCIR (448) comparison between KO and WT mice. Several differentially regulated genes related to cardiovascular development and function, cell–cell signalling and interaction were identified. Therefore, the data obtained from our Flk-1^+/−^ study should serve as a basis for designing future hypothesis driven signalling projects leading to a thorough mechanistic understanding of cardioprotection through VEGF signalling.

## Experimental procedures

### Experimental animals

All animals received care in compliance with the principles of laboratory animal care formulated by the National Society for Medical Research and Guide for the Care and Use of Laboratory Animals published by the National Institutes of Health (NIH). The heterozygous Flk-1 KO (Strain name: B6.129-Kdr^tm1Jrt^/J) mice were purchased from Jackson Laboratory (Bar Harbor, Maine, USA). Restriction maps of the mouse Flk-1 genomic fragment, targeting construct and the structure of the targeted Flk-1 allele is as described [6].

### Experimental protocol

Wild-type (WT) and Flk-1^+/−^ KO mice (male) were randomized into four groups. For Group I (WTIR), after 10 min. stabilization, hearts were perfused for 40 min., followed by exposure to zero-flow normothermic global ischaemia for 30 min. followed by 120 min. of reperfusion (I/R). For Group II (WTPCIR), after stabilization, hearts were subjected to four episodes of 4-min. global ischaemia followed by 6-min. reperfusion before I/R. For Group III (KOIR), hearts were perfused for 40 min. before I/R and for Group IV (KOPCIR), hearts were subjected to the same protocol as WTPCIR.

### Working heart preparation

Mice (25–34 g) were anaesthetized with sodium pentobarbital (150–200 mg/kg body weight IP injection, Abbott Laboratories, Abbott Park, IL, USA) followed by heparin (500 U/kg bw IP injection, Elkins-Sinn Inc., Cherry Hill, NJ, USA) injection. The heart was excised after ensuring sufficient depth of anaesthesia and immediately immersed in ice-cold (4°C) perfusion buffer. The aorta and pulmonary vein were cannulated followed by retrograde perfusion in the Langendorff mode through the aortic cannula was initiated at a perfusion pressure of 60 mm Hg. The perfusion buffer was a modified Krebs-Henseleit Bicarbonate buffer [KHB: composed of (in mmol/l) 118 NaCl, 4.7 KCl, 1.2 MgSO_4_, 25 NaHCO_3_, 10 glucose and 1.7 CaCl_2_, gassed with 95% O_2_:5% CO_2_, filtered through a 5-μm filter to remove any particulate contaminants, pH 7.4] which was maintained at a constant temperature of 37°C and gassed continuously for the entire duration of the experiment [13]. After 10 min. of retrograde perfusion, the heart was switched to antegrade perfusion mode where KHB buffer entered the cannulated left atrium at a pressure equivalent to 10 cm of water, and passed to the left ventricle from which it was spontaneously ejected through the aortic cannula. Control measurements of heart rate, coronary flow, AF, left ventricular developed pressure (LVDP), and its first derivative dp/dt_max_ were monitored, analysed and recorded in real time using the digitized data acquisition and analysis system (Micromed, Louisville, KY, USA). The stabilization procedure in WTPCIR and KOPCIR groups was followed by four short cycles of 4 min. ischaemia and 6 min. of reperfusion in the PC group. The ischaemia reperfusion groups (WTIR and KOIR) underwent a time-matched perfusion. After this period, the hearts in all groups were subjected to 30 min. ischaemia. Before the initiation of 2 hrs reperfusion, the heart was perfused in retrograde mode to avoid the development of high-incidence ventricular fibrillation. The measurements of the cardiac functions were measured out at 30, 60, 90 and 120 min. of the 2 hrs reperfusion period.

### Infarct size

Infarct size (*n*= 6/group) was measured as previously described [12, 13]. After reperfusion, hearts were immediately perfused with 1% (w/v) triphenyltetrazolium chloride. Hearts were excised and stored at −70°C. Sections of frozen hearts were fixed in 10% (v/v) formalin, placed between two coverslips, and digitally imaged with the use of an Epson scanner. To quantitate the areas of interest in pixels, Scion Image (β 4.03 for windows) analysing software was used.

### Determination of cardiomyocyte apoptosis

Formaldehyde-fixed heart tissue sections were embedded in paraffin, cut into transverse sections (4 μm thick), and deparaffinized with a graded series of histoclear and ethanol solutions. Immunohistochemical detection of apoptotic cells was carried out using a TUNEL reaction using *In Situ* Cell Death Detection Kit, Fluorescein as per the manufacturers instructions (Roche Diagnostics, Mannheim, Germany). The sections (*n*= 4) were washed 3× in phosphate buffered saline (PBS), blocked with 10% normal goat serum in 1% bovine serum albumin (BSA) (w/v) in PBS and incubated with anti-α-sarcomeric actin, Sigma) followed by staining with TRITC-conjugated rabbit antimouse IgG (1:200 dilution; Sigma). After incubation, sections were rinsed thrice in PBS and mounted with Vectashield mounting medium (Vector Burlingame, CA, USA). Observed images were captured using a confocal laser Zeiss LSM 410 microscope. For quantitation, the number of TUNEL-positive cardiomyocytes were counted in 100 high-power fields (HPF) [13].

### Microarray analysis

This project was conducted in collaboration with the Cancer Genomics Core facility in the Kimmel Cancer Center, Thomas Jefferson University, Philadelphia, PA, USA. The study was performed with Affymetrix Gene Chip Mouse Genome 430 2.0 array (Affymetrix, Santa Clara, CA, USA). Mouse Genome 430 2.0 array is a single array that contains over 45,000 probe sets representing approximately 34,000 known mouse genes. After completion of the protocols, left ventricular tissue was quickly frozen in liquid nitrogen and stored at −80°C. Frozen left ventricles (*n*= 6/group) were homogenized and DNA-free total RNA was isolated with RNeasy micro kit (Qiagen, Valencia, CA, USA), according to the manufacturers instructions. DNase-treated RNA was ethanol precipitated and quantified on a NanoDrop ND-1000 spectropho-tometer, followed by RNA quality assessment by analysis on an Agilent 2100 bioanalyser (Agilent, Palo Alto, CA, USA). First-strand cDNA was synthesized using Oligo dT and Superscript II RT (Invitrogen, Grand Island, NY, USA). Alternatively, cDNA was prepared using OVATION RNA Amplification System (NuGen Technologies, Inc., San Carlos, CA, USA). cDNA amplification products were fragmented and chemically labelled with biotin to generate biotinylated cDNA targets. Each Affymetrix gene chip for mouse genome 430 2.0 were hybridized with fragmented and biotin-labelled target (2.5 μg) in 200 μl of hybridization cocktail. Target denaturation was performed at 99°C for 2 min., followed by hybridization for 18 hrs. Arrays then were washed and stained using Genechip Fluidic Station 450, and hybridization signals were amplified using antibody amplification with goat IgG (Sigma-Aldrich) and anti-streptavidin biotinylated antibody (Vector Laboratories, Burlingame, CA, USA). Chips were scanned on a Affymetrix Gene Chip Scanner 3000, using GeneChip Operating Software (GCOS) version 3.0. Background correction and normalization were done using Robust Multichip Average (RMA) with Genespring V 7.3.1 software (Silicon Genetics, Redwood City, CA, USA). Volcano plots were used to identify differentially expressed genes using the parametric testing assuming variances are equal (filters based on the results of a Student's two-sample t-test for two groups or a one-way analysis of variance (anova) for multiple groups) and no multiple testing correction. Two different comparisons were done (WTIR *versus* KOIR and WTPCIR *versus* KOPCIR). The differentially expressed gene list was loaded into Ingenuity Pathway Analysis (IPA) 5.0 software (http://www.ingenuity.com) to perform biological network and functional analyses.

### Quantitative real-time RT-PCR

Reverse transcription (RT) was performed with 1 μg total RNA isolated from left ventricular tissue (*n*= 6/group) of WT and Flk-1^+/−^ heterozygous KO mice subjected to I/R with or without PC. Real-time RT-PCR analysis was done with 10 ng of RT product using the iCycler iQ detection system (Biorad, Hercules, CA, USA) employing Syber Green I fluorescence employing β-actin as reference control [12, 13]. Primer sequences used for real-time RT-PCR are given in Table S1 (Supplemental file).

### Statistical analysis

The values for myocardial haemodynamic parameters, infarct size, apoptosis and quantitative real time PCR were all expressed as the mean **±** standard deviation (**±** SD). Differences between groups were tested for statistical significance by one-way analysis of variance (anova) followed by a Bonferroni correction to test for differences between the mean values of all groups with the help of statistical tool (SPSS 15.0). The results were considered significant if *P* < 0.05.

## Results

### Characterization of Flk-1 heterozygous KO mice

**Fig. 1:**
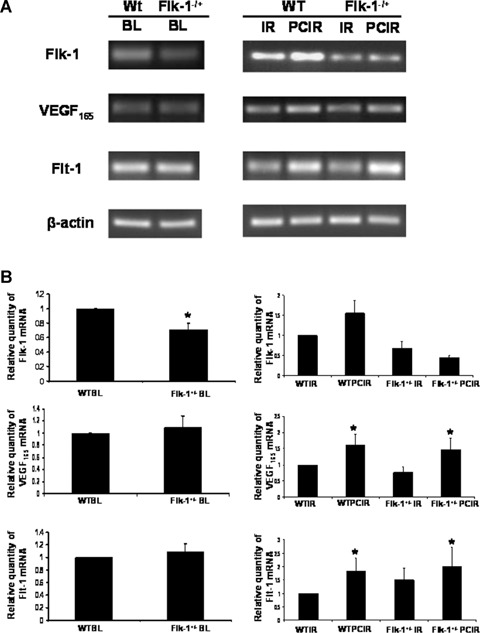
RT-PCR and real-time RT-PCR analysis for Flk-1, Flt-1 and vascular endothelial growth factor (VEGF). (**A**) Relative abundance (%) of Flk-1, Flt-1 and VEGF mRNA in wild-type (WT) and Flk-1^+/−^ knockout myocardium (*n*= 6/group) at the baseline level, after ischaemia/reperfusion (IR) and after ischaemic preconditioning (PCIR) followed by IR. (**B**) Quantitative real-time RT-PCR analysis validating RT-PCR data for VEGF, Flt-1 and Flk-1 mRNA. *P < 0.05 compared with WT ischaemia/reperfusion, #*P* < 0.05 compared with WT preconditioning, †*P* < 0.05 compared with KO ischaemia/reperfusion.

Almost 50% reduction in Flk-1 mRNA was found in hearts from heterozyogous Flk-1 KO mice (Fig. 1A and B) assessed by both RT-PCR and real-time RT-PCR. Moreover, Flk-1 mRNA expression is significantly inhibited in the KOPCIR compared to the WTPCIR myocardium. As expected, expression of Flt-1 and VEGF mRNA are not affected in Flk-1^+/−^ mice before or after I/R (Fig. 1A and B); however, after PC both Flt-1 and VEGF mRNA expression in KOPCIR and WTPCIR were increased compared to I/R.

## Effect of Flk-1 heterozygosity on the recovery of ventricular function after ischaemia reperfusion

**Fig. 2:**
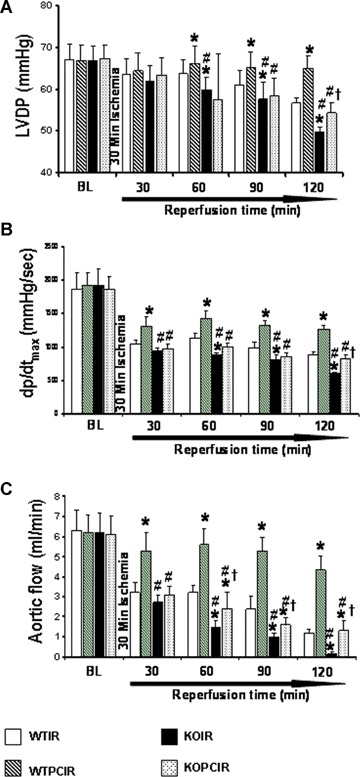
Effects of ischaemia/reperfusion and preconditioning on left ventricular function of wild-type and Flk-1^+/−^ mice. Post-ischaemic ventricular recovery of Flk-1^+/−^ and wild-type mouse hearts (n = 6/group) is presented. The results (**A**) left ventricular developed pressure (LVDP), (**B**) dp/dt_max_ and (**C**) aortic flow are shown in Mean **±** S.D form six animals per group. *P < 0.05 compared with WT ischaemia/reperfusion, #*P* < 0.05 compared with WT preconditioning, †*P* < 0.05 compared with knockout (KO) ischaemia/reperfusion. WTIR, wild-type IR; WTPCIR, preconditioned wild-type, KOIR, Flk-1^+/−^ knockout IR; KOPCIR, preconditioned Flk1^+/−^ knockout.

There was no significant difference in baseline function among the four groups. Throughout the study, the heart rate and coronary flow were not different between the two groups (data not shown). The functional values of each parameter, such as LVDP, dp/dt_max_ and AF, were significantly decreased in all groups after 30 min. of global ischaemia, as expected, compared to their respective baseline values. Post-ischaemic myocardial function was disrupted in the Flk-1^+/−^ mice significantly as evidenced by the significant decrease in LVDP, dp/dt_max_ and AF compared to wild-type control. A significant decrease in LVDP (Fig. 2A) was observed throughout the reperfusion period (except at 30′R). Values after 120 min. of reperfusion for LVDP in KOIR (49.8 ± 1.2) and KOPCIR (54.4 ± 2.6) decreased compared to WTIR (56.8 ±1.1) and WTPCIR (65 ± 3). A significant decrease in dp/dt_max_ (Fig. 2B) also was obtained throughout the reperfusion time after 120 min. of reperfusion in both KOIR (605 ± 13) and KOPCIR (818 ± 55) as compared to the WTIR (884 ± 51) and WTPCIR (1267 ± 51), respectively. Similarly, AF (Fig. 2C) was significantly decreased after 120 min. of reperfusion in both KOIR (0.16 ± 0.1) and KOPCIR (1.3 ± 0.5) compared to WTIR (1.2 ± 0.18) and WTPCIR (4.3 ± 0.72).

### Effect of Flk-1 inhibition on myocardial infarct size

**Fig. 3:**
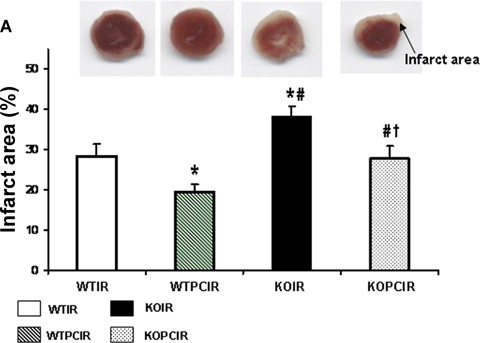

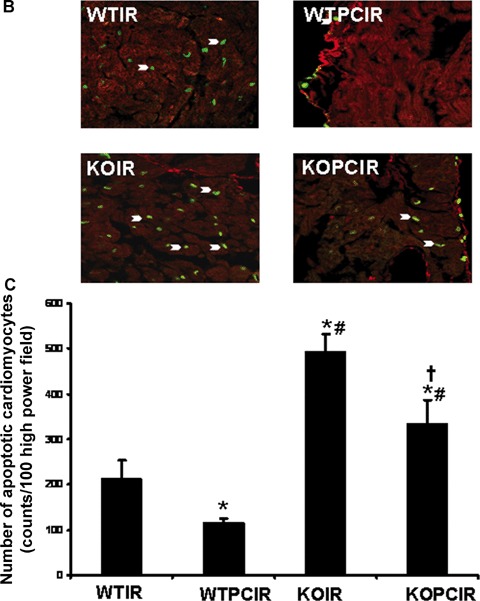
Effects of ischaemia/reperfusion and preconditioning on infarct size and cardiomyocyte apoptosis of wild-type and Flk-1^+/−^ mice. (**A**) Infarct size of the hearts is expressed as a percentage of the area at risk in mouse myocardium subjected to 30 min. of ischaemia followed by 2 hrs of reperfusion. Results are expressed as means **±** SD of six hearts/group. (**B** and **C**) TUNEL assay for apoptotic cardiomyocytes cells after ischaemia/reperfusion and preconditioning of wild-type and Flk-1^+/−^ mice after ischaemia. TUNEL assay for apoptotic cells was performed as described under Experimental procedures. Representative photographs show immunohistochemical staining of extended DNA. Results are expressed as means **±** SEM of six hearts/group. *P < 0.05 compared with wild-type ischaemia/reperfusion group, #*P* < 0.05 compared with Flk^+/−^ ischaemia/reperfusion group, †*P* < 0.05 compared with wild-type preconditioning group. WTIR, wild-type IR; WTPCIR, preconditioned wild-type; KOIR, Flk-1^+/−^ knockout IR; KOPCIR, preconditioned Flk1^+/−^ knockout.

Infarct size expressed as percent infarction relative to total area at risk was noticeably increased in Flk-1^+/−^ mouse hearts compared to controls (Fig. 3A). Transversal cross-sections from Flk-1^+/−^ hearts, which underwent ischaemia reperfusion (38.4%) and ischaemic PC (27.8%) indicated significantly larger (*P* < 0.05) infarct size compared to WTIR (28.41%) and WTPCIR (19.4%) heart sections.

### Effect of Flk-1 inhibition on cardiomyocyte apoptosis by TUNEL Assay

Apoptotic cardiomyocytes (Fig. 3B) were detected using TUNEL staining in conjunction with staining for α-sarcomeric actin. Apoptotic cardiomyocytes were significantly increased in the KOIR (495) and KOPCIR (335) groups when compared to the WTIR (213) and WTPCIR (116) groups (Fig. 3C). Hence, heterozygosity for Flk-1 increased cardiomyocyte cell death due to apoptosis compared to controls. Thus, the extent of cardiac injury is much more prominent in Flk-1^+/−^ KO mice when subjected to ischaemia reperfusion than controls. It is also clear that PC-mediated cardio-protection is disrupted in the KO compared to controls.

### Gene expression changes in the Flk-1 heterozygous and WT mice by Affymetrix Microarray analysis

**Fig. 4:**
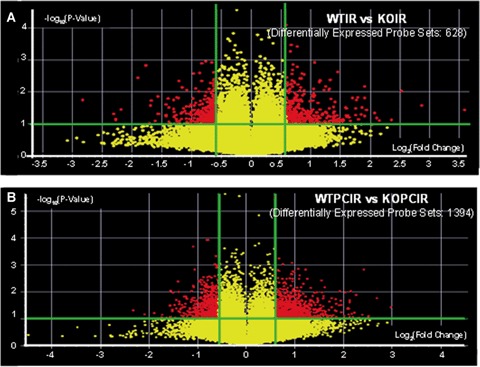
Volcano plot pictures showing the differentially regulated genes in both the comparisions. (**A**) KOIR *versus* WTIR, (**B**) KOPCIR *versus* WTPCIR. Volcano plot was used as filter to view the differentially expressed genes. A volcano plot shows the log_2_(Fold change) in x-axis against the –log_10_(*P*-value) in y-axis. It shows two important measures of differential expression in one plot. Filter genes for a 1.5-fold difference and *P*-value cut-off of 0.1. WTIR, wild-type IR; WTPCIR, preconditioned wild-type; KOIR, Flk-1^+/−^ knockout IR; KOPCIR, preconditioned Flk1^+/−^ knockout.

**Fig. 5:**
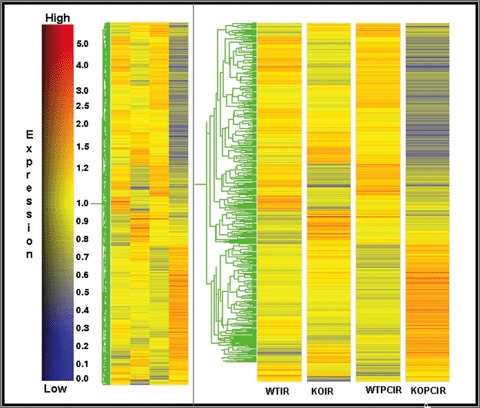
Hierarchic clustering of differentially expressed genes in wild-type and FLK-1^+/−^ mice after Ischaemia reperfusion and ischaemic preconditioning. Data filtering by volcano plot identified 555 genes together in both the comparisons (WTIR *versus* KOIR and WTPCIR *versus* KOPCIR). Gene expression levels are depicted as colour variation from red (high expression) to blue (low expression) The colour in each cell of the figure displays the level of expression for each gene (row) in the myocardium of each group (mean of duplicate valve) (column). WTIR, wild-type IR; WTPCIR, preconditioned wild-type; KOIR, Flk-1^+/−^ knockout IR; KOPCIR, preconditioned Flk1^+/−^ knockout.

Microarray gene profiling was conducted with RNA isolated from the left ventricles of wild-type and KO mice to identify genes involved in ischaemic PC-mediated VEGF signalling. Microarray analysis was performed on RNA isolated from each group to identify differentially expressed genes, using the Affymetrix 430 2.0 mouse genomic array. Signals were loaded into Gene Spring 7.3.1 software and normalized using the RMA algorithm (Supplemental Fig. S1). The differentially expressed genes were further filtered using a Volcano plot (Fig. 4). This plot shows two important measures of differential expression in one plot (−log_10_[*P*-value]*versus* log_2_[Fold change]), allowing decision on which genes are differentially expressed at a particular P-value. The gene expression profile of mouse hearts subjected to PCIR and IR alone in KO mice was compared to the respective PCIR and IR groups in WT mice, that is (WTIR *versus* KOIR and WTPCIR *versus* KOPCIR). The average expression level for each gene was calculated from biological duplicates. Using this Volcano plot at a value *P* < 0.1, showed 628 differentially expressed probe sets in KOIR *versus* WTIR, (Fig. 4A) and 1394 differentially expressed probe sets in KOPCIR *versus* WTPCIR (Fig. 4B). The gene list was reduced to 448 (KOPCIR *versus* WT PCIR) and 115 (KOIR *versus* WTIR) differential expressed genes after applying a cut off intensity 100. A summary of the total number of differentially expressed genes based on a *P*-value <0.1 as shown (Tables 1 and 2). With the goal of discovering specific patterns, hierarchical clustering (Pearson correlation) was applied to the expression profiles of 555 genes (total number of differentially regulated genes). These data are represented by a gene tree (Fig. 5) in which genes of most similar expression patterns are closest to one another. A gene tree or heat map is a graphical means of comparing many genes and samples at one time where each gene and sample is represented by a row and column, respectively. Gene expression (Normalized expression) levels are depicted as colour variation from red (high expression) to blue (low expression).

**Table 1:** Differentially expressed genes in KOIR mice as compared with WTIR mice at different statistical criteria using Volcano plot as a filter

Fold change	*P*-values	Probe sets	100 Cut-off	Unique genes after 100 cut-off
2	0.05	79	18	18
2	0.1	172	34	32
1.5	005	306	56	56
1.5	0.1	628	115	114

**Table 2:** Differentially expressed genes in KOPCIR mice as compared with WTPCIR mice at different statistical criteria using Volcano plot as a filter

Fold change	*P*-values	Probe sets	100 Cut-off	Unique genes after 100 cut-off
2	0.05	195	64	62
2	0.1	419	143	138
1.5	005	646	187	180
1.5	0.1	1394	448	425

A partial list of down-regulated transcripts in WTIR *versus* KOIR comparison (Table 3) and in WTPCIR *versus* KOPCIR (Table 4) is shown. Important genes down-regulated in the KO as compared to WT mice include kinase insert domain receptor (KDR), Syndecan 1 and vascular endothelial cell adhesion molecule-1 (VCAM-1) which plays an important role in cardiovascular function.

**Table 3:** List of selected down-regulated genes in WTIR *versus* KOIR

Affy ID	Gene symbol	Description
1419697_at	CXCL11	Chemokine (C-X-C motif) ligand 11
1437478_s_at	EFHD2	EF hand domain containing 2
1423136_at	FGF1	Fibroblast growth factor 1
1424877_a_at	ALAD	Aminolevulinate, δ-, dehydratase
1434008_at	SCN4B	Sodium channel, type IV, β
1417343_at	FXYD6	FXYD domain-containing ion transport regulator 6
1448304_a_at	RAB6	RAB6, member RAS oncogene family
1449379_at	KDR	Kinase insert domain protein receptor
1452445_at	SLC41A2	Solute carrier family 41, member 2
1436576_at	A630077B13RIK	RIKEN cDNA A630077B13 gene
1424365_at	1810037I17RIK	RIKEN cDNA 1810037I17 gene
1416124_at	CCND2	Cyclin D2 (very important)
1439540_at	MARCH2	Membrane-associated ring finger (C3HC4) 2
1451343_at	VPS36	Vacuolar protein sorting 36
1450036_at	SGK3	Serum/glucocorticoid-regulated kinase 3
1436959_x_at	NELF	Nasal embryonic LHRH factor
1452053_a_at	TMEM33	Transmembrane protein 33
1459783_s_at	CNO	Cappuccino
1434598_at	LARP5	La ribonucleoprotein domain family, member 5
1448780_at	SLC12A2	Solute carrier family 12, member 2
1422464_at	MRPL3	Mitochondrial ribosomal protein L3
1415943_at	SDC1	Syndecan 1
1434577_at	BC052040	cDNA sequence BC052040
1422492_at	CPOX	Coproporphyrinogen oxidase

**Table 4:** List of selected Down-regulated genes in WTPCIR *versus* KOPCIR

Affy ID	Gene symbol	Description
1448183_a_at	Hifla	Hypoxia inducible factor 1, α subunit
1416123_at	Ccnd2	Cyclin D2
1449379_at	Kdr	Kinase insert domain protein receptor
1437284_at	Fzd1	Frizzled homologue 1 (Drosophila)
1415988_at	Hdlbp	High-density lipoprotein (HDL) binding protein
1433972_at	Camtal	Calmodulin-binding transcription activator 1
1420491_at	Eif2s1	Eukaryotic translation initiation factor 2, subunit 1 α
1455396_at	Atp8b1	ATPase, class I, type 8B, member 1 (Atp8b1), mRNA
1428230_at	Prkcn	Protein kinase C, μ
1459457_at	Camk2d	Calcium/calmodulin-dependent protein kinase II, δ
1425354_a_at	Aggfl	Angiogenic factor with G patch and FHA domains 1
1421821_at	Ldlr	Low-density lipoprotein receptor
1423144_at	Pik3ca	Phosphatidylinositol 3-kinase, catalytic, alpha polypeptide
1425512_at	Map2k7	Mitogen-activated protein kinase kinase 7
1424681_a_at	Psma5	proteasome (prosome, macropain) subunit, alpha type 5
1418453_a_at	Atp1b1	ATPase, Na+/K+ transporting, beta 1 polypeptide
1430500_s_at	Mtx2	Metaxin 2
1451090_a_at	Eif2s3x	Eukaryotic translation initiation factor 2, subunit 3, structural gene X-linked
1436003_at	Vcaml	Vascular cell adhesion molecule 1
1460303_at	Nr3c1	Nuclear receptor subfamily 3, group C, member 1
1430990_s_at	Mrpl44	Mitochondrial ribosomal protein L44
1425993_a_at	Hsp110	Heat shock protein 110
1423330_at	Ensa	Endosulfine α
1423456_at	Bzw2	Basic leucine zipper and W2 domains 2
1417204_at	Kdelr2	KDEL (Lys-Asp-Glu-Leu) endoplasmic reticulum protein retention receptor 2
1433641_at	Smad5	MAD homologue 5 (Drosophila)

**Fig. 6:**
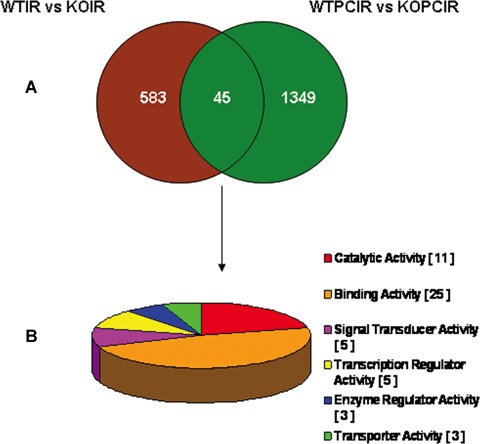
**A** Venn diagram shows the common differentially expressed genes in between two comparisons, (KO I/R *versus* WT I/R and KOPCIR *versus* WT PCIR). (**B**). Pie chart shows the classification of differentially expressed genes based on biological functions (Netaffx gene ontology tool, Affymetrix). The number represents the genes associated with a specific function. Note that some genes may have multiple functions and be classified in several categories. WTIR, wild-type IR; WTP-CIR, preconditioned wild-type; KOIR, Flk-1^+/−^ knockout IR; KOPCIR, preconditioned Flk1^+/−^ knockout.

The Venn diagram in Fig. 6A compares differentially expressed genes in-between the two different comparisons, which showed 45 common genes were differentially regulated. Further classification of the 45 genes according to biological function (Fig. 6B) was determined from the Netaffx gene ontology tool, Affymetrix analysis shows that 11 genes are involved in catalytic activity, 25 genes in binding activity and five in signal transduction activity. A pie chart shows commonly regulated functions (Netaffx gene ontology tool, Affymetrix) for the different comparisons (Fig. 6).

### Biological network and functional analysis

**Fig. 7:**
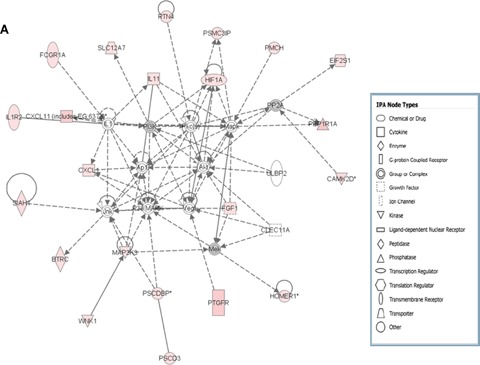

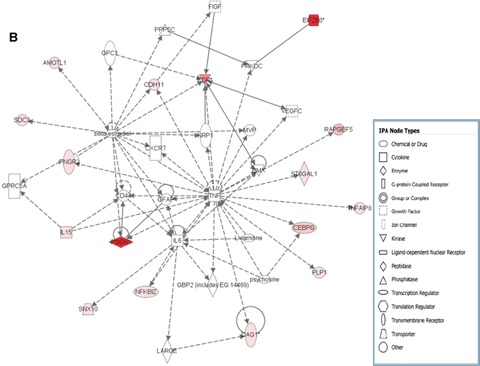
Functionally related gene network constructed from both the comparisons WTIR *versus* KOIR (**A**) and WTPCIR *versus* KOPCIR (**B**) related with cardiovascular system with the help of IPA 5.0.Nodes represent genes, with their shape representing the functional class of the gene product. All the focus genes are represented by pink and the higher intensity red colour represent lower *P*-value WTIR, wild-type IR; WTPCIR, preconditioned wild-type; KOIR, Flk-1^+/−^ knockout IR; KOPCIR, preconditioned Flk1^+/−^ knockout.

In order to further refine the functional properties of the genes down-regulated, the total down-regulated genes in both comparisons (1156 genes) were loaded in the IPA tool for the network, functional and pathway analysis. IPA converts a list of genes (with or without accompanying expression information) into a set of relevant networks based on the ingenuity pathways knowledge base (IPKB). Total down-regulated genes (1156 transcripts) in both comparisons were selected and used for network generation and functional analysis. Affymetrix probe ID and *P*-value were loaded into the IPA software. A core analysis is performed for the list of genes in individual groups, which was followed by comparison analysis (WTIR *versus* KOIR and WTPCIR *versus* KOPCIR). The genes were categorized based upon molecular function in the IPA software. The identified genes were also mapped to genetic networks in the IPA database and ranked by score. The score reflects the probability that a collection of genes equal to or greater than the number in a network could be achieved by chance alone. A score of more than 10 was used as a cut-off for identifying gene networks. The list of networks generated (Table 5 and 6) and important networks related to cardiovascular function in each comparison is given (Fig. 7A and B). Network analysis revealed two important genetic networks related to cardiovascular development, which is shown in Fig. 7A (WTIR *versus* KOIR) and 7B (WTPCIR *versus* KOPCIR). Two networks had a high score of 42 and 26 with 22 and 16 focus genes, respectively. Several important genes, such as AKT, AP1, FGF1, HIF1A, IL1, MAP3k3, MEK, P38MAPK, VEGF, Eif2s3, KDR etc, are related in the network. Further functional and pathway classification showed, several genes down-regulated related to organismal injury and abnormalities, cardiovascular system development and function, free radical scavenging, cellular growth and proliferation, skeletal and muscle disorders in both comparisons. Table 7 and 8 show the list of functions with respect to the *P*-value and related number of genes in WTIR *versus* KOIR and WTPCIR *versus* KOPCIR comparisons.

**Table 5:** Selected genetic networks with high scores (>10) in WTIR *versus* KOIR comparison

Molecules in network	Score	Focus genes	Top functions
Akt, Ap1, BTRC, CAMK2D, CLEC11A, CXCL1, CXCL11 (includes EG:6373), EIF2S1, FCGR1A, FGF1, HIF1A, H0MER1, IL1, IL11, IL1R2, Jnk, MAP3K3, Mapk, Mek, P38 MAPK, PI3K, Pkc(s), PMCH, PP2A, PPP1R1A, PSCD3, PSCDBP, PSMC3IP, PTGFR, RTN4, SIAH1,SLC12A7, ULBP2, Vegf, WNK1	42	22	Cardiovascular system development and function, cell-to-cell signalling and interaction, gene expression
Amino acids, ARHGDIG, ARHGEF11, B3GALT2, CASC3, CD38, CDC6, CDC25C, CENPJ, COG2, COG7, CPE, GYS1 (includes EG:2997), HNRPA1, HNRPC, hydrogen peroxide, INS1, MAGOH, MCF2L, MPO, NXF1, PHGDH, PLK3, P0LR2A, P0U2F1, PPM1D, PTPRO, RBM8A, RCN1, RHOA, RHPN2, SYTL4, THOC4, TNF, TNFAIP8	27	15	Carbohydrate metabolism, lipid metabolism, molecular transport
ARFGEF1, ARHGDIG, ARHGEF11, β-estradiol, CAD, CD200, CD200R1, CDH4, CLCN3, CRSP2, CUBN, DOK1, EGF, FOXA2, HCRTR2, IGHMBP2, LDLR, Mmp, MMP2, MY09B, PDGFB, PKIB, PLCE1, PSCD3, RHOA, SCARB1, SERPINA1, SETD7, SGK3, SLC9A3R1, TAF7, TBN, TBP, TRFP, USP6NL	25	15	Lipid metabolism, molecular transport, small molecule biochemistry
AK3L1, ATR, CCNA1, CCNA2, CD9, CDC6, CDC25A, CDC25B, CDC25C, CDK6, CHEK2, COL18A1, CPOX, E2F6, E2F1 (includes EG:1869), EGLN1, ERCC3, FBXW11, FLU, GTF2H1, HIF1A, MGA (includes EG:23269), MXD4, MYCT1, NDNL2, NRN1, PRKDC, RBX1, SREBF1, STARD4, TGFB1, TP53, USP7, ZFP161, ZNF22	24	14	Cell cycle, gene expression, DNA replication, recombination and repair
AHR, CLEC11A, CYP2B6, DLG2, DLG3, DLGAP1, DNAJC11, FLNB, FOS, GRASP, GRIN1, heparin, HLTF, IL10, MAGI2, MLLT10, MMP2, MPG, NRP1, PCNX, POLR3A, POLR3F, PPARBP, RB1, RBBP9, retinoic acid, SE -MA3D, SHANK2, SMARCA4, S MAR -CB1, SPTBN1, ST6GALNAC4, TCOF1 (includes EG:6949), YY1, ZFAND5	25	15	Organismal development, gene expression, cancer

**Table 6:** Selected genetic networks with High scores (>10) in WTPCIR *versus* KOPCIR comparison

Molecules in network	Score	Focus genes	Top functions
ADRBK2, BTRC, CCND2, Creb, DRD1, DYRK2, FCGR2B, FLU, GRIP1, HDC, HIF1A, HIF1AN, HLTF, IL11, Jnk, Mapk, MAPT, NCAM1, NPR3, P38 MAPK, Pdgf, PI3K, Pkc(s), PMCH, PRKG1 (includes EG:5592), RUNX -1, Scf, SLC6A3, SP1, TCF12, Tgf beta, TLR4, Vegf, WNT5A,YES1	45	24	Cell-to-cell signalling and interaction, cellular growth and proliferation, cellular development
Akt, Ap1, APAF1, CXCL11 (includes EG: 6373), DUSP6, FLI1, FST, Hsp90, Jnk, LDL R, Mapk, NR3C1, P38 MAPK, p70 S6k, Pdgf, PDGFC, PEPCK, PI3K, PIK3C3, PIK3CA, PMCH, PP1/PP2A, PPARGC1A, PPM1L, PPP1R11, PSCDBP, PSMC3IP, RASSF1, Scf, SULF1, TFDP1,Tgf-β, TLR3, TRAF3, VCAM1	40	22	Endocrine system development and function, lipid metabolism, molecular transport
AM0TL1, β-estradiol, BMX, CD44, CDH11, CEBPG, CXCR7, DAG1, EIF2S3, FIGF, GBP2 (includes EG:14469), GFAP, GPC1, GPRC5A, IFNGR2, IL6, IL15, KDR, L-carnitine, LARGE, MVP, NFKBIZ, NRP1, PLP1, PPP5C, PRKDC, PSEN1, psychosine, RAPGEF5, SDC3, SNX10, ST6GAL1, TNF, TNFAIP8, VEGFC	26	16	Cellular movement, cardiovascular system development and function, organismal development
ARCN1, ATM/ATR, BCL10, BRCA1, BRCC3, BRE, CASP9, CCND2, CHEK2, DAG1, ELK1, EPOR, GAST, HDC, HMGN3, IL3, IL1RL1, KLF7, MDC1, PCDH7, PLK3, PTX3, RAD50, RANBP9, RAPGEF5, RELB, ROB01, SRPK2, TNF, TNFAIP3, TNFAIP6, TNFAIP8, TROVE2, USP11, ZFAND5	26	16	Cell death, cell cycle, cancer
ATR, Caspase, CDH1, CDT1, CEP55, CUL4B, ERCC1, ETS1, FAM3C, Groucho, HAS2, HNRPA2B1, HRAS, HRASLS, IGFBP3,IGSF4(includesEG:23705), JMJD1C, MATN4, MLLT4, NOTCH1, P8, PAXIP1, PVR, PVRL- 3, RUNX1, SOAT1, SON, TIMP3, TLE1, TP53, TP53BP1, TRIM44, UPP1, UTY, Zn2+	24	15	Cell death, connective tissue disorders, cancer
ATXN1, AURKA, CDC7, CEP55, CHGN, DBF4, DDX6 (includes EG:1656), EP400, GAPDH (includes EG:2597), GPS2, GSR, JMJD1C, KIAA1267, MAP4, MARK4 (includesEG:57787), MCM2, MCM4, MCM6, MCM7, MED6, MGA (includes EG:23269), MLL, PPP1R15A (includes EG:23645), PRNP, PVRL3, SFRS10, TBL1X, TFDP1, THPO, TNFRSF10A (includes EG:8797), TP53, TRIM44, TXNRD1, USP7,WDR5	24	15	DNA Replication, Recombination and repair, cell cycle, cell death

**Table 7:** Gene ontology analysis of the genes affected in WTIR *versus* KOIR comparison

Relevant functions	P-value	No. of molecules
Organismal injury and abnormalities	8.39E-4 – 4.76E-2 10	10
Cardiovascular system development and function	8.39E-4 – 4.47E-2 9	9
Free radical scavenging	7.59E-3 – 7.59E-3 1	1
Cellular growth and proliferation	7.59E-3 – 4.95E-2 23	23
Haematological system development and function	7.59E-3 – 4.95E-2 14	14
Molecular transport	7.59E-3 – 4.57E-2 12	12
Cellular development	7.59E-3 – 4.47E-2 7	7
Connective tissue development and function	7.59E-3 – 4.47E-2 6	6
Drug metabolism	7.59E-3 – 4.47E-2 6	6
Cellular movement	7.59E-3 – 4.47E-2 19	19
Cell cycle	7.59E-3 – 3.74E-2 7	7
Nucleic acid metabolism	7.59E-3 – 3.74E-2 2	2
DNA replication, recombination and repair	7.59E-3 – 3.00E-2 4	4
Metabolic disease	7.59E-3 – 3.00E-2 4	4
Cardiovascular disease	6.18E-3 – 4.47E-2 8	8
Tissue development	5.89E-4 – 4.90E-2 22	22
Skeletal and muscular system development and function	5.89E-4 – 4.47E-2 10	10
Cell death	4.89E-3 – 4.76E-2 11	11
Embryonic development	4.89E-3 – 4.47E-2 4	4
Cell-to-cell signalling and interaction	4.61E-3 – 4.47E-2 19	19
Cellular assembly and organization	3.58E-3 – 4.47E-2 21	21
Skeletal and muscular disorders	3.58E-3 – 4.47E-2 11	11
Nutritional disease	3.00E-2 – 3.74E-2 1	1
Protein trafficking	2.26E-2 – 3.00E-2 2	2
Organismal development	1.58E-3 – 4.57E-2 15	15
Vitamin and mineral metabolism	1.51E-2 – 4.57E-2 4	4
Cell signalling	1.51E-2 – 4.57E-2 19	19
Post-translational modification	1.51E-2 – 3.74E-2 4	4
Cellular function and maintenance	1.51E-2 – 2.26E-2 5	5
Gene expression	1.21E-3 – 4.91E-2 27	27
Lipid metabolism	1.17E-3 – 4.47E-2 6	6

**Table 8:** Gene ontology analysis of the genes affected in WTPCIR *versus* KOPCIR comparison

Relevant functions	P-value	No of Molecules
Cellular compromise	9.02E-3 – 4.43E-2 7	7
Lipid metabolism	9.02E-3 – 4.43E-2 6	6
Organismal injury and abnormalities	9.02E-3 – 4.43E-2 6	6
Cardiovascular disease	9.02E-3 – 4.43E-2 4	4
Organismal development	9.02E-3 – 4.43E-2 4	4
Carbohydrate metabolism	9.02E-3 – 4.43E-2 3	3
Endocrine system disorders	9.02E-3 – 4.43E-2 3	3
Small molecule biochemistry	9.02E-3 – 4.43E-2 23	23
Respiratory system development and function	9.02E-3 – 4.43E-2 2	2
Molecular transport	9.02E-3 – 4.43E-2 12	12
Skeletal and muscular system development and function	9.02E-3 – 4.43E-2 11	11
Skeletal and muscular disorders	9.02E-3 – 4.38E-2 9	9
Tissue development	9.02E-3 – 4.38E-2 12	12
Organ Development	9.02E-3 – 3.56E-2 6	6
DNA replication, recombination, and repair	9.02E-3 – 3.56E-2 5	5
Cardiovascular system development and function	9.02E-3 – 2.68E-2 4	4
Cell Signalling	8.21E-3 – 4.43E-2 5	5
Cellular assembly and organization	7.08E-6 – 4.43E-2 17	17
Embryonic development	6.85E-3 – 4.43E-2 9	9
Cell death	6.85E-3 – 4.43E-2 8	8
Cellular growth and proliferation	4.49E-4 – 4.43E-2 15	15
Cell-to-cell signalling and interaction	4.49E-4 – 4.43E-2 13	13
Organismal survival	3.02E-2 – 4.11E-2 15	15
Vitamin and mineral metabolism	2.68E-2 – 4.43E-2 2	2
Amino acid metabolism	2.44E-2 – 4.43E-2 9	9
Post-translational modification	2.44E-2 – 4.43E-2 8	8
Protein trafficking	2.03E-2 – 2.03E-2 5	5
Cellular function and maintenance	1.80E-2 – 4.43E-2 6	6
Tissue morphology	1.65E-3 – 4.43E-2 16	16
Gene expression	1.18E-3 – 4.43E-2 10	10

### Validation of differentially expressed genes by realtime RT-PCR

**Fig. 8:**
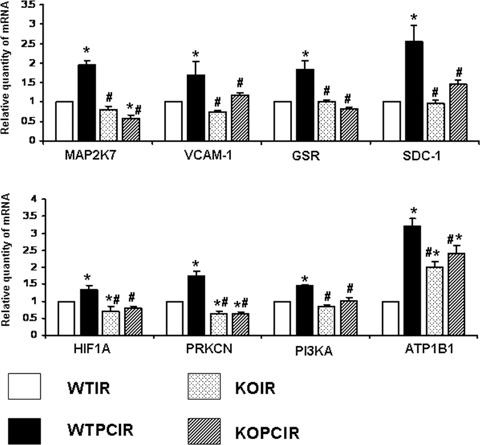
Independent validation of down-regulated genes by real-time RT-PCR. *P < 0.05 compared with wild-type ischaemia/reperfusion group, *P < 0.05 compared with WT ischaemia/reperfusion, #*P* < 0.05 compared with WT preconditioning, †*P* < 0.05 compared with KO ischaemia/reperfusion. WTIR, wild-type IR; WTPCIR, preconditioned wild-type; KOIR, Flk-1^+/−^ knockout IR; KOPCIR, preconditioned Flk1^+/−^ knockout.

Real-time RT-PCR analysis was performed to confirm the relative expression patterns of randomly chosen down-regulated genes in both comparisons. The four experimental groups (WTIR, KOIR, WTPCIR and KOPCIR) were assessed for each transcript. This approach led to successful verification of nine transcripts involved in VEGF-mediated cardioprotection through Flk-1 signalling during ischaemic PC and include VCAM, HIF-1A and mitogen-activated protein kinase kinase 7 (Fig. 8). The mRNA levels of VCAM, HIF-1A, mitogen-activated protein kinase kinase 7 along with other genes increased in PC groups as compared with the IR groups in both KO and WT mice. However, the levels were significantly decreased in KO mice. Real time RT-PCR analysis revealed results consistent with the microarray data, thus demonstrating the accuracy of the array approach

## Discussion

This study explores for the first time how ischaemic PC-mediated cardioprotection modifies genomic responses significantly in Flk-1 heterozygous receptor KO mice and disrupts PC-mediated cardioprotection. Expression of VEGF RTKs, such as Flk-1 and Flt-1 in endothelial cells, has made it difficult to document individual contributions of each receptor to VEGF signalling. This study shows that ischaemia/reperfusion and / or PC modifies genomic responses in Flk-1^+/−^KO myocardium compared to WT, specifically, modifying expression of genes responsible for myocardial response to ubiquitination, angiogenesis and survival, etc. that are obviously important for cardioprotection. In the current study, we used genetically manipulated heterozygous Flk-1 (50% down-regulated) KO mice because homozygous KO mice are not viable due to early embryonic death caused by inhibition of vasculogenesis [10].

As assessed by RT-PCR and real-time RT-PCR, VEGF and Flt-1 mRNA expression in KO mice, compared to WT mice, are not affected at the baseline level by disruption of the Flk-1 gene. However, after PC, both VEGF and Flt-1 mRNAs are significantly up-regulated in WTPCIR and KOPCIR compared to their respective IR groups. On the other hand, as expected Flk-1 mRNA was down-regulated (50%) at the baseline level as well as in the KOPCIR group. These observations indicate that disruption of Flk-1 gene does not affect PC-mediated up-regulation of VEGF and Flt-1 mRNA. Again, functional recovery after PC deteriorates in Flk-1^+/−^ compared to wild-type mice. Infarct size is also found to be greater in the KOPCIR (27.8%) than in the WTPCIR (19.4%) group; and therefore, PC-mediated cardioprotection is diminished significantly even though these are heterozygous KO mice with 50% of Flk-1 receptors still available for VEGF-medi-ated signalling.

In this study, we have confirmed that PC-induced VEGF signalling is disrupted by knocking down Flk-1, and is characterized by a decrease in haemodynamics, an increase in infarct size and apoptosis. Several high-throughput technologies have been used to investigate the effect of ischaemic PC-mediated myocardial changes [14, 15]; but, to our knowledge, this is the first microar-ray study of global gene expression in Flk-1^+/−^ mice using high-density oligonucleotide microarrays. Left ventricular RNA samples from six mice in each group were analysed for differential expression by hybridizing with Affymetrix 430 2.0 Mouse arrays. Our data uncovered several pathways induced by VEGF signalling through Flk-1 and provide a framework for comparing whole-heart gene expression changes associated with ischaemic PC.

Gene chip data analysis with the help of Genespring software analysis revealed 448 genes and 115 genes differentially expressed in WTIPCIR *versus* KOPCIR and WTIR and KOPCIR comparisons which showed the involvement of several genes related with ischaemic PC-mediated VEGF-Flk-1 signalling. Identification of the differentially expressed genes and cluster analysis [16] of these genes are the important initial steps, but further analysis of these genes by IPA software for networks/functional analysis allowed us to look into more informative and convincing evidence of changed biological processes due to ischaemic PC. Pathway analysis showed several genes such as presenelin -1, AKT, AP1, FGF1, HIF1A, IL1, MAP3k3, MEK, P38MAPK, VEGF, Eif2s3, KDR related to WNT, FGF, PI3 kinase, cardiac β adrenergic, VEGF and platelet-derived growth factor signalling were down-regulated in the KO mice as compared with the WT mice during ischaemic PC. Hence, the pathway analysis proved that knocking down Flk-1 showed disruption of several important signalling mechanisms related to myocardial angiogenesis.

To independently validate array data, eight down-regulated genes related to myocardial angiogenesis and cardioprotection were selected randomly followed by qRT-PCR analysis. The genes include syndecan-1, Na^+^/K^+^ATPase transporting, β 1 polypeptide, phosphatidylinositol 3-kinase, catalytic, α polypeptide, protein kinase Oμ, mitogen-activated protein kinase kinase 7, vascular cell adhesion molecule 1, hypoxia inducible factor-1, α subunit and glutathione reductase (GR).

Syndecan 1 is a cell surface proteoglycan and an integral membrane protein acting as receptors for the extracellular matrix. Syndecan 1 has been proven to modulate the WNT pathway [17], which is important in cell signalling. Na^+^/K^+^ ATPase activity in the myocardium plays an important role in generating the rapid upstroke of the action potential and drives several ion exchange and transport processes crucial for normal cellular functions [18].

In addition, phosphatidylinositol 3-kinase is found in the down-regulated list, and is composed of 85 kD and 110kD subunits. The 85 kD subunit lacks PI3-kinase activity and acts as an adaptor coupling the 110 kD subunit (p110) to activated protein tyrosine kinases. Phosphatidylinositol 3-kinase, catalytic, α polypeptide has been reported to play an important role in VEGF-mediated angiogenesis [19]. Homozygous KO for this gene leads to embryonic lethality. Hetrozygous mice were viable and fertile [20], but showed severely blunted signalling *via* insulin receptor substrate (IRS) proteins, which is a key mediator of insulin, IGF1 and leptin action. Evidence also suggested [21] a critical role for p110α in growth factor and metabolic signalling. Our study shows that p110α plays an important role in several signalling pathways, including myocardial angiogenesis.

Another intriguing gene on this list was Protein Kinase Cμ. Protein kinase D (PKD)/protein kinase Cμ[22, 23] and two recently identified serine protein kinases termed PKD2 and PKC/PKD3, are similar in overall structure and primary amino acid sequence to PKD [23–25] and constitute a new protein kinase subfamily separate from the previously identified PKCs.

Interestingly, two important genes (VCAM 1 and HIF-α) were identified as down-regulated. HIF-1 (hypoxia-inducible factor-1) is a heterodimer consisting of HIF-1α and HIF-Iβ subunits. The regulation of HIF-1 activity is mainly through the HIF-1α subunit. Translation of HIF-1α to the nucleus and heterodimerization of the α and β subunits demonstrates adaptive responses to ischaemia [26] in the myocardium promoting angiogenesis by activating target genes such as VEGF, LDH and several other genes.

VCAM-1 is a member of the immunoglobulin superfamily of adhesion molecules [27], and is expressed in fibroblast-like cells. The intensity of VCAM-1 expression correlates with the degree of inflammation. The interaction of VCAM-1 and its ligands, the inte-grins, may play significant role in angiogenesis. Pathway analysis also demonstrated down-regulation of GR which is a homodimeric flavoprotein which maintains the cellular thiol redox state by catalyzing the reduction of glutathione disulpide (GSSG) to glutathione. Its activity is present in both the cytosol and mitochondria [28].

In conclusion, use of gene chip technology allowed us for the first time to identify several target genes downstream of VEGF/Flk-1 signalling in PC myocardium. Moreover, biological network and pathway analysis revealed several other related genes indirectly affected due to down-regulation of directly affected genes in the KO. Finally, to our knowledge, this is the first report in VEGF receptor-2 KO mice (Flk-1^+/−^) in which several important genes related to cardioprotection and angiogenesis have been documented and should help to facilitate the design of effective future therapies.

## Supplementary material

The following supplementary material is available online for this article:

**Fig. S1** Scheme of the micorarray protocol.

**Table S1** Primer pairs designed for RT-PCR

This material is available as part of the online article from: http//www.blackwellsynergy.com/doi/abs/10.1111/j.1582-4934.2008.00269.x

(This link will take you to the abstract).
